# Role of Homologous Fc Fragment in the Potency and Efficacy of Anti-Botulinum Antibody Preparations

**DOI:** 10.3390/toxins9060180

**Published:** 2017-05-29

**Authors:** Amram Torgeman, Eyal Ozeri, Alon Ben David, Eran Diamant, Osnat Rosen, Arieh Schwartz, Ada Barnea, Arik Makovitzki, Avishai Mimran, Ran Zichel

**Affiliations:** Department of Biotechnology, Israel Institute for Biological Research, Ness Ziona 7410001, Israel; amit@iibr.gov.il (A.T.); eyalo@iibr.gov.il (E.O.); alonb@iibr.gov.il (A.B.D.); erand@iibr.gov.il (E.D.); osnatr@iibr.gov.il (O.R.); ariehs@iibr.gov.il (A.S.); adabarnea@gmail.com (A.B.); arikm@iibr.gov.il (A.M.); avishaim@iibr.gov.il (A.M.)

**Keywords:** botulinum, antitoxin, potency, efficacy, monoclonal antibodies, polyclonal antibodies

## Abstract

The only approved treatment for botulism relies on passive immunity which is mostly based on antibody preparations collected from hyper-immune horses. The IgG Fc fragment is commonly removed from these heterologous preparations to reduce the incidence of hyper-sensitivity reactions. New-generation therapies entering the pipeline are based on a combination of humanized monoclonal antibodies (MAbs), which exhibit improved safety and pharmacokinetics. In the current study, a systematic and quantitative approach was applied to measure the direct contribution of homologous Fc to the potency of monoclonal and polyclonal antitoxin preparations in mice. Homologous Fc increased the potency of three individual anti-botulinum toxin MAbs by up to one order of magnitude. Moreover, Fc fragment removal almost completely abolished the synergistic potency obtained from a combined preparation of these three MAbs. The MAb mixture neutralized a 400-mouse median lethal dose (MsLD_50_) of botulinum toxin, whereas the F(ab′)_2_ combination failed to neutralize 10 MsLD_50_ of botulinum toxin. Notably, increased avidity did not compensate for this phenomenon, as a polyclonal, hyper-immune, homologous preparation lost 90% of its potency as well upon Fc removal. Finally, the addition of homologous Fc arms to a heterologous pharmaceutical anti-botulinum toxin polyclonal horse F(ab′)_2_ preparation improved its efficacy when administered to intoxicated symptomatic mice. Our study extends the aspects by which switching from animal-based to human-based antitoxins will improve not only the safety but also the potency and efficacy of passive immunity against toxins.

## 1. Introduction

Antibodies (Abs) are the most prevalent among new pharmaceuticals approved in the last two decades [1]. Most of the newly approved Abs consist of human or humanized monoclonal antibodies (MAbs) with high specificity to a molecule that plays an essential role in a specific metabolic pathology. However, in the case of infectious diseases, an individual MAb is rarely sufficient to enable neutralization of a whole bacterium, virus or a toxin molecule [2,3]. Thus, for over a century, passive immunity against infectious agents was based on polyclonal antibody (PAb) preparations made from the sera of hyper-immunized animals. Although PAb preparations were efficient, these polyclonal preparations raised safety concerns due to potential anaphylactic reactions associated with the systemic administration of foreign proteins in high concentrations [4,5]. Elimination of the Fc fragment from these heterologous polyclonal preparations improved their safety [6]. Recent studies suggest that a combination of individual MAbs to create an oligoclonal preparation exhibits synergistic increased potency that may be comparable to that of PAb preparations, paving the way for the use of MAbs as a valid alternative to animal sera-based Ab preparations for passive immunity (reviewed in [7]).

Botulinum neurotoxins (BoNTs), produced by *Clostridium botulinum* strains, are considered the most lethal known toxins, with an estimated human median lethal dose (HLD_50_) of 1 ng/kg body weight [8,9,10,11]. Seven immunological BoNT serotypes are known (A–G), of which types A, B and E (and rarely F) are responsible for most cases of human botulism [12].

Standard therapy for botulism includes administration of equine polyclonal antitoxin and, in severe cases, intensive support care by means of mechanical ventilation [13,14,15,16,17]. Development of “second-generation” antitoxins is now underway [18,19,20]. These preparations are based on combinations of either human-origin or humanized MAbs that are intended to facilitate improved pharmacokinetics along with reduced potential side effects associated with the injection of heterologous horse Abs [5,18,20].

A fundamental advantage of human or humanized MAbs over animal-based polyclonal preparations is the presence of homologous Fc fragments. The two most important mechanisms by which homologous Fc is thought to exert therapeutic effects are improved pharmacokinetics of the Ab, primarily by protection from degradation through binding the neonatal Fc receptor (FcRn), and enhanced clearance of antigen-Ab complexes from the circulation [21,22,23,24,25,26,27]. Recently, the involvement of the Fc fragment in neutralization was demonstrated in mice by increasing the valency of anti-BoNT, heterologous camelid Abs with homologous Fc fragments, by means of genetic engineering [27,28]. In another work, Mazuet and colleagues compared the full IgG and F(ab′)_2_ forms of two anti-BoNT mAbs. In spite of the clear longer half-life that was demonstrated for the intact IgG in blood, the impact on neutralization efficacy was inconclusive [29]. Hence, a comprehensive direct comparison of intact IgG and its F(ab′)_2_ fragment counterpart in the context of potency and efficacy still needs further elucidation, particularly in view of the differences between homologous and heterologous preparations.

In the present study, we further elucidated the direct contribution of homologous Fc fragment to Ab-mediated toxin neutralization by systematic and quantitative evaluation of the potency and efficacy of intact and Fc-removed anti BoNT MAb and PAb preparations. The experimental design was based on the comparison of the F(ab′)_2_ fragment and the corresponding IgG forms of individual and combined preparations of anti-botulinum MAbs in mice and the differential neutralization potencies measured for homologous and heterologous hyper-immune PAb preparations in both in vivo and in vitro systems. Our results clearly demonstrate the substantial contribution of the Fc fragment to the potency of individual MAbs, with even greater effects on the synergistic neutralization potency of MAb combinations. Moreover, homologous Fc was shown to be essential for in vivo but not in vitro optimal neutralization by PAb preparations, and the addition of a homologous Fc arm to a heterologous pharmaceutical horse F(ab′)_2_ fragment preparation improved neutralization efficacy when the fragment preparation was administered to intoxicated symptomatic mice.

## 2. Results

### 2.1. Individual Homologous Intact MAbs Exhibit Increased Neutralizing Activity Compared to Their F(ab′)_2_ Fragment Counterparts

To examine the role of the Fc fragment in homologous Ab neutralization, F(ab′)_2_ fragments were prepared from three individual mouse anti-BoNT/A MAbs and directly compared to their respective IgG molecules for the evaluation of neutralizing activity. The intact IgG forms of these MAbs (A-1, A-2 and A-6) were previously reported to neutralize BoNT/A [23]. To compare the direct neutralization effects, the binding activity of the generated F(ab′)_2_ molecules was first evaluated in comparison to their respective IgG form by ELISA. When equimolar concentrations of each IgG and the respective F(ab′)_2_ fragment were tested, similar titers were measured (IgG/F(ab′)_2_ titer ratios ranging from 0.8–1 (Table 1), confirming that functional binding was not impaired by Fc fragment removal.

**Figure 1:**
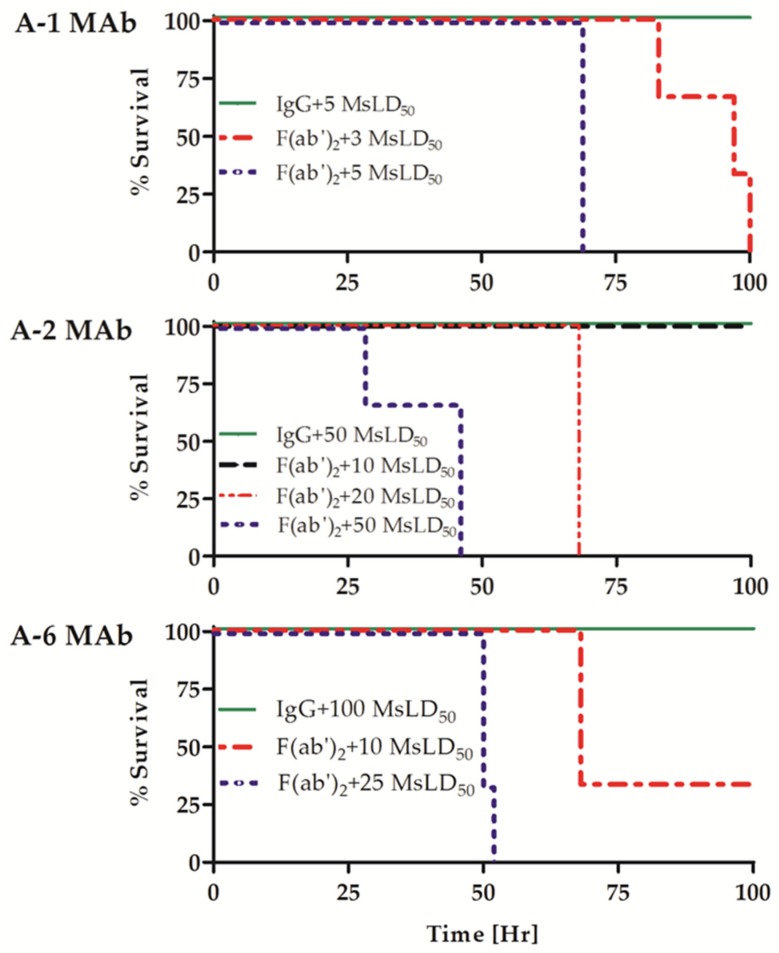
Protective activity of MAb IgG and F(ab′)_2_ fragments in mice. Fixed concentrations of three individual IgG MAbs or their corresponding F(ab′)_2_ fragments were pre-incubated with the indicated BoNT/A toxin doses and then injected into mice (*n* = 3). Survival was monitored for four days, and the percentage survival of each treatment group is shown.

To compare the potency of the Ab forms, equimolar amounts of each IgG MAb or the corresponding F(ab′)_2_ fragment were incubated with increasing concentrations of BoNT/A as indicated in Figure 1. The mixtures were then injected into mice, and survival was monitored for four days. As shown in Figure 1, the A-6 clone IgG completely neutralized 100 MsLD_50_, while only a subset of the mice treated with its F(ab′)_2_ fragment survived a 10 MsLD_50_ toxin dose challenge. None of the mice challenged with 25 MsLD_50_ in the F(ab′)_2_ A-6 clone group survived (*p* = 0.022). Similarly, the intact IgG MAb clone A-2 protected 100% of mice from a challenge of up to 50 MsLD_50_ BoNT/A, whereas its F(ab′)_2_ fragment failed to neutralize toxin dose of 20 MsLD_50_ (*p* = 0.025) and protected against only 10 MsLD_50_ of the toxin. The A-1 IgG protected against 5 MsLD_50_, and administration of the A-1 F(ab′)_2_ fragment failed to neutralize this toxin dose (*p* = 0.025) and resulted in only partial protection of mice exposed to 3 MsLD_50_ toxin. The exact determination of the difference between the potency of the A-1 forms could not be completed due to the proximity of the potency to the assay limit of detection (1 MsLD_50_). Nevertheless, the results clearly show that in contrast to the ~1 IgG/F(ab′)_2_ fragment-binding ratios measured by the ELISA, the potency of individual F(ab′)_2_ fragments in mice was significantly lower (between 5- to 10-fold and possibly even higher for A-1 and A-2 MAbs) than that of their IgG forms (Table 1).

### 2.2. Fc Is Essential for the Synergistic Neutralizing Activity of Oligoclonal MAb Preparations

**Figure 2:**
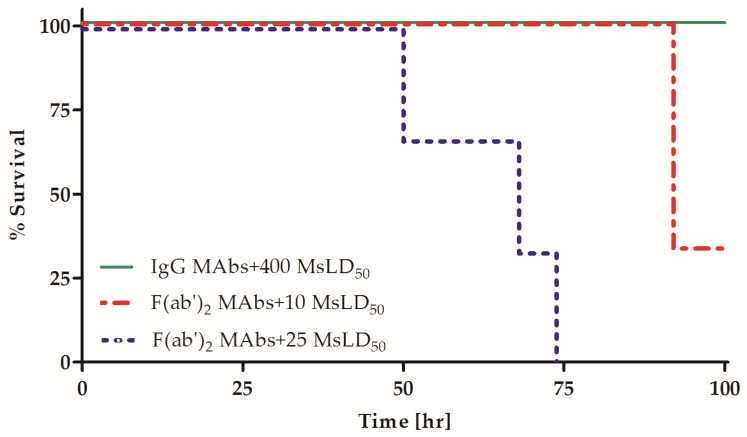
Fc removal abolishes the synergistic potency of an IgG MAb combination. Mixtures of three MAb IgGs or F(ab′)_2_ fragments were pre-incubated with the indicated BoNT/A concentrations and then injected into the mice (*n* = 3). Survival was monitored for four days.

The combination of several homologous MAbs was previously demonstrated to synergistically increase their potency in vivo [22,23,30]. Although the Fc fragment was suggested to play a role in this phenomenon, the direct effect of the Fc fragment on the synergistic combination of anti-BoNT homologous MAbs has not been assessed. To directly elucidate the role of homologous Fc fragments in the synergistic neutralization of an anti-BoNT oligoclonal preparation, the potency of a combination of the three MAbs presented in Figure 1 was compared between both the F(ab′)_2_ fragment and IgG forms. Fixed, equimolar amounts of MAbs, either IgG or F(ab′)_2_ fragments, were combined and incubated with increasing concentrations of BoNT/A. The mixtures were then injected into mice, and survival was monitored. In accordance with the expected increased potency of the MAb combination, IgG and F(ab′)_2_ fragment preparations were used at a 1:10 dilution of the concentration used when tested individually in this set of experiments (Figure 2). MAb potency was confirmed to be linear over this dilution range (Supplementary Table S1). The combined IgG preparation neutralized 400 LD_50_, a 25-fold higher potency than the expected additive effect based on individual neutralization (15.5 MsLD_50_ after a 1:10 dilution, Figure 2 and Table 2). In contrast, the combined F(ab′)_2_ fragment preparation only partially neutralized 10 MsLD_50_, a potency value that is less than 4-fold higher than the expected additive effect (less than 2.3 MsLD_50_ with a 1:10 dilution; Figure 1). Thus, the potency of the combined IgG preparation was almost two orders of magnitude higher than that of its F(ab′)_2_ fragment counterpart (*p* = 0.02). Notably, the intact IgG and F(ab′)_2_ fragments equally bound to their target antigen in ELISA at the concentrations tested in vivo. These results directly demonstrate that the presence of Fc fragments is essential for not only individual MAb potency, but also the pronounced synergistic effect conferred by a combined IgG MAb mixture.

### 2.3. The Potency of a Homologous But Not a Heterologous Hyper-Immune, PAb Preparation Is Dependent on the Presence of Fc Fragments

It is reasonable to hypothesize that in contrast to the pronounced effect on MAbs, the potency of hyper-immune PAb preparations will be less affected by the elimination of homologous Fc fragments, since the avidity of such preparations is expected to compensate for the differences in potency observed among the MAbs. To directly assess the role of homologous Fc fragments in the potency of PAbs and to expand the repertoire of botulinum serotype tested, murine and equine hyper-immune anti-BoNT/B preparations were tested for their neutralization properties in both the intact IgG and the F(ab′)_2_ forms. To this end, mouse anti-Hc/B (homologous PAbs) and horse anti-BoNT/B (heterologous PAbs) preparations were measured for their neutralizing antibody concentration in vivo using the European Pharmacopoeia reference method. To exclude possible differences due to titer and binding activity, PAb preparations were normalized according to their functional binding to their target protein by ELISA (Supplementary Table S2). The mouse intact IgG PAb preparation had at least an 8-fold higher neutralization potency over its F(ab′)_2_ fragment counterpart (8 IU/nmol and <1 IU/nmol, respectively, Table 3). Thus, the elimination of the Fc fragment from the homologous, hyper-immune PAb preparation led to nearly a one order of magnitude reduction in its neutralization potency. In contrast, when the heterologous PAb preparation (Horse anti BoNT/B) was tested in the same set-up, the potency of the intact IgG and its F(ab′)_2_ fragment were similar (15 IU/nmol and 14 IU/nmol, respectively). These results suggest that homologous Fc fragments are essential for fully enhancing the neutralization potency of both MAb and hyper-immune PAb preparation.

### 2.4. Homologous Fc Increases the Potency and Efficacy of a Heterologous Anti-BoNT PAb Preparation

**Figure 3:**
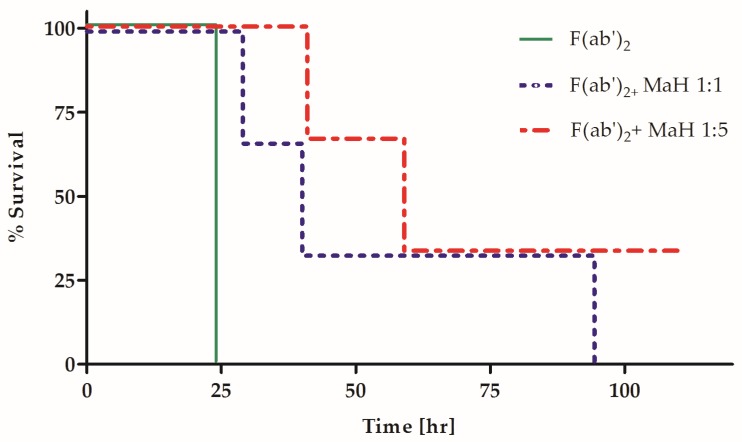
Homologous Fc fragment improves the efficacy of a heterologous antitoxin in vivo. Three groups of mice (*n* = 3) were treated with either 0.1 IU/Mouse of horse polyclonal F(ab′)_2_ fragment alone or with addition of mouse anti-Horse F(ab′)_2_ (MaH) in various molar ratios (1:1 or 1:5) 13 h after intoxication with 4 MsLD_50_ BoNT A. Survival was monitored for five days.

It is very likely that the lack of impaired potency in Fc-eliminated horse PAbs resulted from the inability of the heterologous equine Fc fragment to interact in vivo with the mouse Fc-mediated machinery. Thus, we hypothesized that the addition of homologous Fc fragments to the heterologous F(ab′)_2_ preparation would result in improved potency. Since molecular engineering techniques are not applicable for the addition of homologous Fc fragments to a polyclonal hyper-immune horse Ab preparation, a mouse hyper-immune PAb preparation was generated against the horse F(ab′)_2_ fragment. In this manner, the mouse IgG anti-horse F(ab′)_2_ served as a homologous Fc arm that was introduced to mice treated with the equine F(ab′)_2_ antitoxin. Mice were exposed to an intramuscular injection of 4 MsLD_50_ of BoNT/A and were then treated at the indicated time points post-exposure with 0.1 IU of the horse anti BoNT/A F(ab′)_2_ fragment preparation. Fifteen minutes after the administration of horse anti-BoNT, mice were divided into three groups (*n* = 3) and treated either with a 1:5 (group no. 1) or 1:1 (group no. 2) molar ratio of a mouse anti-horse F(ab′)_2_ fragment PAb. The third group of mice served as a control and was not treated with the mouse anti-horse F(ab′)_2_ fragment PAb preparation. Survival was monitored for five days (Figure 3). The administration of a homologous anti-horse IgG significantly delayed the time to death in mice treated with the horse anti-BoNT F(ab′)_2_ fragment. The delayed time to death was observed at 7, 13 and 15 h post-exposure, with the most pronounced effect at 13 h in both Ab ratios (*p* = 0.025). The ELISA binding experiments confirmed that the mouse anti-horse F(ab′)_2_ PAb did not interfere with BoNT binding by the horse F(ab′)_2_ preparation (Supplementary Figure S1). When the same experimental setup was applied to measure the neutralizing Ab concentration using the standard Pharmacopeia assay, the mouse anti-horse F(ab′)_2_ increased the potency of the horse anti-BoNT F(ab′)_2_ preparation by up to 20%, from 600 to 800 IU/mL. Thus, the addition of a homologous Fc to a pharmaceutical heterologous F(ab′)_2_ preparation led to significant increases in both potency and efficacy.

### 2.5. The Contribution of Homologous Fc to Neutralization Potency Depends on In Vivo Interaction with Host-Expressed Components

The improved potency and efficacy measured for intact IgG over its F(ab′)_2_ fragment in homologous but not heterologous Ab preparations suggested that homologous Fc interacts in vivo with host components as part of the mechanism that governs improved protection. A possible way to demonstrate this interaction is by comparing the potency of the IgG and F(ab′)_2_ forms in an in vitro neutralization assay. We recently developed an in vitro neutralization potency assay that highly correlated with the Pharmacopeial mouse neutralization assay [31]. The assay was used to determine the potency of anti-BoNT pharmaceutical PAb preparations and is expected to reduce the requisite use of animals for the approval of such preparations. To test the role of in vivo host components in toxin neutralization by homologous antitoxins, the potency of the mouse anti-Hc/B PAb was tested using the in vitro potency assay in both the intact IgG and the F(ab′)_2_ fragment forms.

The potency of mouse PAb F(ab′)_2_ fragment was 18.8 (±1) IU/nmol, while the potency of original IgG preparation was 21 (±2) IU/nmol. Thus, the significantly improved potency measured in vivo for the full IgG form of the mouse preparation (8 vs. <1 IU/nmol for the IgG and F(ab′)_2_ fragments, respectively) was almost completely abolished when the Fc-interacting component was absent from the in vitro neutralization reaction.

## 3. Discussion

The IgG forms of three individual anti-BoNT MAbs (A-1, A-2 and A-6) conferred significantly improved protection over their respective F(ab′)_2_ fragment forms. Intact MAbs and their F(ab′)_2_ fragments were administered in equimolar concentrations in respect to their binding titer to rule out any bias as a result of differences in functional binding. Our observations are not entirely consistent with results reported by Mazuet et al., who conducted a BoNT toxicity study with two MAbs; one F(ab′)_2_ fragment exerted less protection in mice, while the other F(ab′)_2_ fragment retained the neutralization efficacy of its intact IgG, despite the clearly longer half-lives for both intact IgGs in the blood [29]. Interestingly, the MAb that was not affected by the Fc fragment deletion in the Mazuet et al. study displayed a neutralization activity that was comparable to that of a PAb preparation [29]. It is very rare for a single MAb to be as protective as a PAb-based preparation. A high-affinity MAb reflects a stronger binding to the target toxin despite the reduced serum half-life and may present a unique state in which the F(ab′)_2_ fragment MAb is removed from the blood while tightly bound to BoNT toxin, thereby compensating for the expected reduction in pharmacokinetics.

Consistent with our results, Pincus and colleagues showed that low doses of an anti-ricin intact MAb provided significantly better protection in mice than the corresponding F(ab′)_2_ fragment [32]. Since we have shown that the F(ab′)_2_ fragment binding activity is not altered and since Fc-mediated clearance is not considered to occur in individual IgG MAbs [26,30,33], it is reasonable to argue that the reduced potency of the single F(ab′)_2_ fragment that we and others have observed may be attributed to a shorter half-life.

Despite the advantageous presence of homologous Fc fragments, the potency of the majority of MAbs remains relatively low when compared to PAb-based preparations, especially those of hyper-immune origin. However, the combined preparation of 2–3 homologous MAbs presents synergistic neutralization that may be compared to that of polyclonal preparations [7]. Nonetheless, to the best of our knowledge, the contribution of the Fc fragment in anti-BoNT synergistic neutralization has not been directly elucidated. In the current study, removal of the Fc fragment from a combined preparation consisting of three anti-BoNT MAbs almost completely abolished their capacity to demonstrate synergistic potency. The combined F(ab′)_2_ fragment preparation failed to protect against a 10 MsLD_50_ toxin dose, whereas the intact IgG combined counterpart neutralized 400 MsLD_50_. Interestingly, the combined F(ab′)_2_ molecule preparation did present some extended potency over the expected additive effect (~2.3 MsLD_50_). However, the difference from the expected potency was marginal (less than 4-fold), and it is difficult to determine whether it expresses Fc fragment-independent marginal synergy or merely an empiric variation between expected and actual additive potency of the F(ab′)_2_ fragment combined preparation. Clearly, in each of these possibilities, these results demonstrate a canonical role for the Fc fragment in the synergistic neutralization of BoNT by a homologous anti-toxin IgG MAb cocktail in vivo. Moreover, these results suggest that protection by heterologous equine antitoxin requires a higher dose of the F(ab′)_2_ molecule to compensate for the lack of Fc-mediated mechanisms. However, the elevated amount of foreign protein administered to a patient raises the risk for side effects.

Chow SK and co-workers studied synergistic neutralization of a MAb combination consisting of two anti-*Bacillus anthracis* protective antigen (PA) Abs and have shown that this phenomenon was dramatically altered in transgenic mice in which the gene for the Fc fragment receptor was knocked out [34]. In accordance with the observation of Chow and colleagues, our results provide direct evidence for the role of the homologous Ab Fc fragment in this phenomenon. We assume that decoration of the toxin with increased valence Fc, which leads to enhanced clearance of MAb-toxin complexes from the serum, rather than improved pharmacokinetics, was the primary mechanism governing the synergism [27,28,33,35]. The potency of the IgG MAb cocktail preparation was improved considerably, not only over their combined F(ab′)_2_ fragments preparation, but also over the expected additive potency of the three individual intact MAbs that were already shown to present Fc-mediated extended pharmacokinetics. Thus, while the presence of Fc is essential for a longer half-life of a single MAb, several toxin-bound Fc moieties are required to induce the synergistic protection of a combined IgG MAb mixture. Indeed, we were previously able to demonstrate that addition of non-neutralizing intact MAb to an anti-BoNT MAb combination significantly contributed to the synergistic effects, exerted [23].

We hypothesized that eliminating the Fc fragment from a polyclonal, homologous, hyper-immune preparation would result in a negligible effect on its potency due to the expected dominant effect of avidity. Nevertheless, the intact anti-BoNT IgG PAb preparation lost 90% of its potency after removal of its Fc fragment. This dramatic loss of potency was not observed with the heterologous Ab preparation, as both equine IgG and F(ab′)_2_ forms of an antitoxin exhibited similar potencies in mice. The dependence of this phenomenon on the interaction between homologous Fc and host molecules, presumably FcRs which in turn exerts Fc-dependent clearance mechanism, was confirmed by measuring equal potency for the "homologous" mouse intact IgG and F(ab′)_2_ fragment PAbs in an in vitro neutralization assay. This assay consists of the machinery for intoxication but, for obvious reasons, lacks the involvement of cellular Fc-mediated functions. In good agreement with these findings, mice were equally protected when treated with intact or F(ab′)_2_ fragment horse PAb anti-ricin [32]. Nevertheless, we were able to demonstrate that enrichment of a horse F(ab′)_2_ fragment anti-BoNT preparation with a homologous Fc fragment arm, in the form of a hyper-immune mouse PAb anti-horse F(ab′)_2_ fragment, significantly improved its efficacy when administered to symptomatic mice 13 h after exposure to BoNT. Overall, our results demonstrate that specific interactions of the homologous Fc fragments with the host Fc-binding components are essential for conferring optimal passive protection by MAbs and MAb combinations as well as polyclonal hyper-immune preparations.

The inter-species specificity of the Fc fragment to its receptors may have significant implications for the measurement of the potency of pharmaceutical antitoxin preparations [21,36,37]. Mouse FcRn binds human and mouse Fc fragments with similar affinity [24]. In contrast, human FcRn binds human Fc fragments with a much higher affinity than that for mouse Fc fragments [24]. This difference means that the current mouse potency assay approved by the Pharmacopeia to measure the potency of equine botulinum antitoxins is valid and will reflect the full potency of combined MAb preparations bearing human Fc. Indeed, Nowakowski et al. demonstrated synergistic toxin neutralization in mice by combining recombinant full IgG MAbs consisting of murine V-domains and human C-domains [30]. Inter-species specificity may also explain the differences in potency Jones et al. have found between heterologous anti BoNT IgG PAb and its fragments in systemic toxicity [38]. This inter-species specificity also stresses that the humanization of mouse MAbs is essential not only to reduce immunogenicity, which is less of a concern in single-use treatment against infectious agents, but also to confer higher compatibility with the Fc receptor, which will significantly extend its efficacy in patients.

In conclusion, the current study provides extended insights to the contribution of Fc fragments to toxin neutralization exerted by MAb and PAb preparations. The study emphasizes the significance of promoting the approval of human-derived antitoxins [18,19,39,40,41,42] as safe and potent alternatives to traditional animal-based preparations.

## 4. Materials and Methods

### 4.1. Ethics Statement

All animal experiments were performed in accordance with Israeli law and were approved by the Ethics Committee for Animal Experiments at the Israel Institute for Biological Research (research permit number M-24-2014). All efforts were made to minimize suffering. During the survival studies, loss of righting reflex was used as the humane end-point of the experiment. Mice were monitored three times a day for their condition and for the occurrence of end-point. Mice that presented loss of righting reflex were humanely euthanized.

### 4.2. Materials

All chemicals were purchased from Sigma-Aldrich (Rehovot, Israel) unless otherwise stated. Recombinant receptor binding domains of botulinum neurotoxin type A (Hc/A) and type B (Hc/B) were produced and purified as previously described [43,44]. Mouse anti-H_C_/A MAbs were prepared as previously described [23]. Mouse anti-H_C_/B and horse anti BoNT/B PAbs were purified from the sera of hyper-immune animals that had been immunized with H_C_/B or BoNT/B, respectively, as previously described [45]. Mouse anti-horse F(ab′)_2_ fragment PAbs were collected from hyper-immune mice as previously described [23].

### 4.3. Toxins

*Clostridium botulinum* A and B strains were obtained from the Israel Institute for Biological Research (IIBR) collection (A198 and B592, respectively). Sequence analysis revealed compliance of the neurotoxin genes with serotypes 62A (GenBank Accession Number M30196) and Danish (GenBank Accession Number M81186) of *C. botulinum* types A1 and B1, respectively [46,47]. Toxins complex were prepared from concentrated supernatants of cultures grown for 6 days in anaerobic culture tubes. Toxins A and B used had specific activity of 7.4 × 10^6^ and 1.5 × 10^7^ MsLD_50_/mg, respectively. BoNTs were handled in a Class II bio-safety cabinet (Thermo Fisher Scientific, Waltham, MA, USA). Each toxin stock was incubated in 50 mM citrate buffer (pH = 5.5) at −70 °C. Toxins were further diluted to a working stock concentration in gelatin buffer (0.2% w/v gelatin in phosphate buffer, pH = 6.4). For determination of the lethal dose (mouse LD test), different doses of the toxin were injected intraperitoneally into groups of mice (*n* = 5) as described by Malizio et al. [48] and lethality was calculated according to Spearman–Karber method [49].

### 4.4. IgG Purification and Generation of F(ab′)_2_ Fragments

Mouse Abs were purified using a protein A column (GE Healthcare, Uppsala, Sweden). Mouse anti-horse F(ab′)_2_ fragment-specific Abs were obtained using direct affinity chromatography, with an NSH resin (GE Healthcare, Uppsala, Sweden) conjugated to purified horse F(ab′)_2_ fragment (Jackson ImmunoResearch Laboratories Inc., West Grove, PA, USA). To generate anti-F(ab′)_2_ fragments, an F(ab′)_2_ fragment preparation kit (Thermo Scientific, Rockford, IL, USA) was used according to the manufacturer’s instructions. The relative purity of the F(ab′)_2_ fragment preparations was evaluated to be >90% by Coomassie blue staining of proteins subjected to non-reducing Sodium dodecyl sulfate polyacrylamide gel electrophoresis (SDS-PAGE).

### 4.5. IgG and F(ab′)_2_ Fragment Quantification Assay

Total IgG and F(ab′)_2_ fragment protein concentrations were determined by sandwich ELISA using a specific set of Abs for mouse and horse preparations. Briefly, for mouse MAbs and PAbs (IgG and F(ab′)_2_ fragment preparations), microtiter plates (MAxisorp, Nunc, Roskilda, Denmark) were coated with 100 ng (50 µL/well) of goat anti-mouse F(ab′)_2_ fragment-specific Abs (Jackson ImmunoResearch Laboratories Inc., West Grove, PA, USA). For horse IgG PAbs, 100 ng (50 µL/well) of goat anti-horse IgG Fc fragment-specific Abs (Jackson ImmunoResearch Laboratories Inc., West Grove, PA, USA) was used. For horse F(ab′)_2_ fragment quantification, plates were coated with 100 ng (50 µL/well) of goat anti-horse F(ab′)_2_ fragment-specific Abs (Jackson ImmunoResearch Laboratories Inc., West Grove, PA, USA). All coatings were conducted with coating buffer (50 mM Na_2_CO_3_, pH 9.6) and then incubated overnight at 4 °C. The plates were then washed in wash solution (WS, 0.9% NaCl, 0.05% Tween 20) and blocked for 1 h at 37 °C with 200 µL per well of 2% (w/v) bovine serum albumin (BSA) in Tris-NaCl pH 7.6 (TSTA). Following blocking and washing, plates were incubated with serial dilutions (50 µL, in duplicate) of the IgG or F(ab′)_2_ fragment samples in TSTA (50 µL/well, in duplicate) for 1 h at 37 °C. After an additional wash with WS, plates were incubated with 1:500 (50 µL/well) alkaline phosphatase-conjugated goat anti-mouse Fab-specific Abs (for mouse MAbs and PAbs) (Sigma-Aldrich, Rehovot, Israel) or with goat anti-horse F(ab′)_2_ fragment-specific Abs (for horse PAbs) (Jackson ImmunoResearch Laboratories Inc., West Grove, PA, USA). All conjugated Abs were diluted in TSTA. Following incubation for 1 h at 37 °C, plates were washed with WS, and the color reaction was developed using *p*-nitrophenyl phosphate substrate (1 mg/mL in 0.2 M Tris buffer). Absorbance was measured at 405 nm with a Molecular Devices Spectramax M3 reader. The IgG and F(ab′)_2_ fragment Ab concentrations were determined by interpolation from a 1.56–100 ng/mL standard curve (fitted to a four-parameter equation) of the respective mouse and horse ChromPure IgG whole molecule or F(ab′)_2_ fragment (Jackson ImmunoResearch Laboratories Inc., West Grove, PA, USA) using SoftMax Pro software 5.4 (Molecular Devices, Sunnyvale, CA, USA).

### 4.6. Determination of Anti-BoNT IgG and F(ab′)_2_ Fragment Titers by ELISA

ELISA plates were coated with 120 ng/well of Hc/A (for MAbs) or 250 ng/well of Hc/B (for mouse PAbs) diluted in coating buffer. After an overnight incubation at 4 °C, the plates were washed three times with WS and blocked for 1 h at 37 °C with TSTA. After an additional washing step, serial dilutions of the tested Ab preparations in a final volume of 50 μL were added and incubated 1 h at 37 °C. For each Ab preparation, the equimolar concentration of the intact IgG and its respective F(ab′)_2_ fragment was used as the starting dilution. The plates were then washed four times, an alkaline phosphatase-conjugated goat anti-mouse Fab-specific Ab (Sigma-Aldrich, Rehovot, Israel) diluted 1:500 in TSTA (for MAbs) was added, and the plates were incubated for 1 h at 37 °C. The plates were washed four times, and the colorimetric reaction was developed using the substrate *p*-nitrophenyl phosphate (50 µL/well). Finally, absorbance was measured at 405 nm, and titers were determined as the last dilution with a signal greater than three standard deviations above the background signal generated with TSTA buffer.

### 4.7. MAb In Vivo Potency Assay

Equimolar concentrations of IgG and F(ab′)_2_ fragment of each MAb were diluted in gelatin buffer (0.2% w/v gelatin in phosphate buffer, pH = 6.4) and incubated with an equal volume of solution containing different concentrations of BoNT/A for 1 h at 25 °C. In the MAb combined experiments, each IgG or F(ab′)_2_ fragment was further diluted by 1:10 beyond the respective dilutions in the individual MAb potency assay due to expected synergistic effects. Each toxin/Ab mixture was injected intraperitoneally (I.P.) into CD-1 female mice (Charles River UK) (3 mice, 1 mL per mouse), and survival was monitored for four days. A MAb was considered neutralizing if 100% survival was achieved.

### 4.8. PAbs In Vivo Potency Assay

The neutralizing Ab concentration was determined according to European Pharmacopoeia [50]. Briefly, serial 1.2-fold dilutions of an antitoxin preparation were prepared. Simultaneously, a standard antitoxin preparation was diluted to the final concentrations of 0.08, 0.10, 0.12, 0.14 International Units per mL (IU/mL). All antitoxin dilutions were then mixed with a toxin test dose of 2200 MsLD_50_, previously calibrated according to the Pharmacopoeia, and the mixtures were incubated for 1 h at 25 °C. Each mixture was injected I.P. into four mice (CD-1; Charles River UK) (1 mL per mouse), and survival was monitored for four days. Antitoxin potency was calculated based on the lowest dilution of antitoxin that failed to protect the animals when compared to that of the standard antitoxin.

### 4.9. In Vitro Neutralization Assay

The in vitro neutralization assay was performed as previously described [31]. Dilutions of the BoNT/B and the antitoxins were identical to those described in the in vivo assay section. The additional standard antitoxin dilutions used were 0.04 and 0.06 IU/mL. All antitoxin dilutions were incubated for 1 h at 25 °C with a toxin test dose of 2200 MsLD_50_. Then, each toxin/antitoxin mixture (1 mL) was incubated while shaking at 800 rpm with 40 µL of Syt-II40-60-conjugated magnetic beads for 1 h at 37 °C. Next, the supernatant was removed, and the beads were washed three times with decreasing volumes (1, 0.5, 0.2 mL) of PBS containing 0.1% BSA and 0.025% Tween, followed by three additional washing steps with 0.2 mL of PBS (after the second wash with PBS, the beads were transferred to a new tube). After the final washing step, the supernatant was removed, and the beads were tested for toxin activity using the Endo-Peptidase assay.

Endo-peptidase activity was measured as previously described [51,52,53,54,55]. Briefly, the reaction was performed in a 20-µL reaction volume containing toxin-bound beads, 100 µM peptide substrate, 1 mM ZnCl_2_, 1% Triton, 10 mM dithiothreitol (DTT), and 50 mM HEPES buffer (pH 7.3) at 37 °C with shaking for 5 h. One hundred eighty microliters of 1% formic acid was added, and the samples were then incubated for 2 min at 100 °C. Beads were removed prior to ultra-high-performance liquid chromatography (UPLC) analysis.

### 4.10. UPLC Analysis

A 10-µL aliquot of each reaction supernatant was analyzed with a Waters Acquity UPLC (Waters Corporation, Milford, MA, USA) equipped with a UV detector and binary solvent manager. The output signal was monitored and processed using Empower software (Empower 2.0, Waters Corporation, Milford, MA, USA). The method was employed using an Acquity UPLC BEH C18 1.7 µm (2.1 × 50 mm) column (Waters Corporation, Milford, MA, USA) as described previously [31]. The flow rate of the mobile phase was 0.15 mL/min. The column temperature was 50 °C, and the eluted products were monitored at a wavelength of 215 nm. The cleaved products were rinsed for 3 min in an acetonitrile gradient from 95% buffer A (5% acetonitrile in 0.1% trifluoroacetic acid [TFA]) and 5% buffer B (80% acetonitrile in 0.1% TFA) to 70% buffer A. Quantitative analysis of the products was performed by calculating the area of the product peak using a standard calibration curve. Unless stated otherwise, all UPLC equipment was from Waters (Waters Corporation, Milford, MA, USA), and materials were from Sigma-Aldrich (Rehovot, Israel).

### 4.11. Statistical Analysis

A four-parameter logistic regression model was used to construct a standard curve in all ELISA assays using SoftMax Pro 5.4 (Molecular Devices, Sunnyvale CA, USA). Comparisons of survival curves were performed with the log-rank (Mantel-Cox) test using GraphPad Prism 5 software (La Jolla, CA, USA). Differences were considered significant when *p* < 0.05.

## Figures and Tables

**Table 1:** ELISA titers and potencies of IgG and F(ab′)_2_ fragments of the anti-BoNT/A MAbs.

Antibody	Normalized ELISA Titer per 1 µM Antibody			Neutralizing Activity ^a^		
	IgG	F(ab′)_2_	IgG/F(ab′)_2_ Ratio	IgG [MsLD_50_]	F(ab′)_2_ [MsLD_50_]	IgG/F(ab′)_2_ Ratio
A-6	3.6 × 10^4^	4.4 × 10^4^	0.8	100	<10	>10
A-2	2.2 × 10^5^	2.2 × 10^5^	1.0	50	10	5
A-1	3.1 × 10^4^	3.5 × 10^4^	0.9	5	<3	>1.7

^a^ Data represent the highest toxin doses that were neutralized in vivo. MsLD_50_ = median lethal dose; BoNT = Botulinum neurotoxin; MAb = monoclonal antibodies.

**Table 2:** Fc is essential for the synergistic neutralizing activity of oligoclonal MAb preparations.

A-1 + A-2 + A-6 MAb Combination	Expected Neutralization [MsLD_50_] ^a^	Measured Neutralization [MsLD_50_]	Synergism ^b^
IgGs	15.5	400	~25
F(ab′)_2_	2.3	<10	<4

^a^ Equimolar amounts of IgGs and F(ab′)_2_ fragments were used. MAb concentrations were 1/10 of the concentrations used when tested individually (Figure 1). Expected values are based on the calculated additive potency at 1/10 of the individual protection results. ^b^ Measured/Expected neutralization ratio.

**Table 3:** Potency of IgG and F(ab′)_2_ fragment PAbs in mice.

PAb Source	Neutralizing Activity in Vivo		
	IgG [IU/nmole]	F(ab′)_2_ [IU/nmole]	IgG/F(ab′)_2_ Ratio
Mouse	8	<1	<8.00
Horse	15	14	1.07

## Supplementary Materials

The following are available online at www.mdpi.com/2072-6651/9/6/180/s1, Table S1: Linear neutralizing activity of MAbs cocktail, Table S2: ELISA titers of IgG and F(ab′)_2_ fragments of the anti-BoNT/B PAbs, Figure S1: Addition of a mouse antibody arm to horse F(ab′)_2_ anti-BoNT/A preparation does not affect its toxin binding activity.
