# Supplementary material for: Role of Homologous Fc Fragment in the Potency and Efficacy of Anti-Botulinum Antibody Preparations
Source: Toxins (Basel). 2017 May 29;9(6):180. doi: 10.3390/toxins9060180 (PMC5488030; doi:10.3390/toxins9060180)
Supplement: Supplementary file 1 [file toxins-09-00180-s001.pdf]

Supplementary Materials: Role of Homologous Fc Fragment in the Potency and Efficacy of Anti-Botulinum Antibody Preparations

Amram Torgeman, Eyal Ozeri, Alon Ben David, Eran Diamant, Osnat Rosen, Arieh Schwartz, Ada Barnea, Arik Makovitzki, Avishai Mimran and Ran Zichel

Table S1. Linear neutralizing activity of MAbs cocktail.

MAb Cocktail Dilution	Neutralized Toxin Dose [$MsLD_{50}$]
1:100	6.4×10^4
1:1000	6.4×10^3

Cocktail consisting of seven MAbs including A-1, A-2 and A-6.

Table S2. ELISA titers of IgG and $F(ab')_2$ fragments of the anti-BoNT/B PAbs.

PAb Source	Normalized ELISA Titer per 1 μM Antibody		
	IgG	$F(ab')_2$	IgG/$F(ab')_2$ Ratio
Mouse	4.9×10^4	2.6×10^4	1.90 [a]
Horse	6.9×10^3	6.8×10^3	1.01

[a] Neutralizing activity was normalized accordingly.

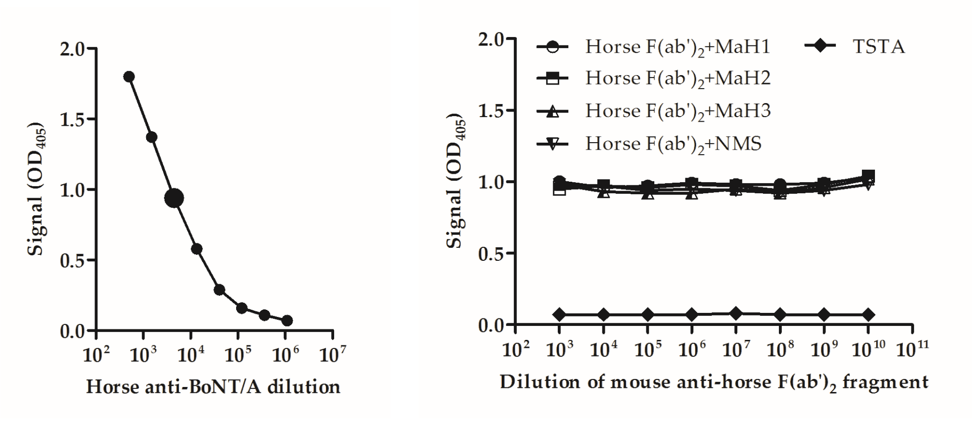

(**a**) (**b**)

Figure S1. Addition of a mouse antibody arm to horse $F(ab')_2$ anti-BoNT/A preparation does not affect its toxin-binding activity. (**a**) Plates were coated with BoNT/A and the indicated dilutions of horse $F(ab')_2$ anti-toxin were tested for their binding. Dilution of 4.5×10^3 (marked with a large circle) yielding an OD of ~1 was selected to be tested in the competition assay presented in panel b. (**b**) Competition assay between horse $F(ab')_2$ anti-BoNT/A and Mouse anti-horse $F(ab')_2$ antibody preparations. Plates were coated with BoNT/A and the indicated dilutions of mouse anti-horse $F(ab')_2$ antibody (MaH) from three individual mice (MaH-1,2,3) were pre-incubated with a constant dilution of anti-BoNT/A horse $F(ab')_2$, prior to their addition to the plate. Naïve Sera (Normal mouse serum - NMS) was used as a control. TSTA buffer indicates the background signal.
